# Exploring the views and experiences of callers to the PANDA Post and Antenatal Depression Association Australian National Perinatal Depression Helpline: a cross-sectional survey

**DOI:** 10.1186/s12884-015-0594-0

**Published:** 2015-09-07

**Authors:** Laura J. Biggs, Touran Shafiei, Della A. Forster, Rhonda Small, Helen L. McLachlan

**Affiliations:** Judith Lumley Centre, La Trobe University, Level 3, 215 Franklin St, Melbourne, 3000 Australia; The Royal Women’s Hospital, Locked Bag 300 Grattan St & Flemington Rd, Parkville, VIC 3052 Australia; School of Nursing & Midwifery, La Trobe University, Cnr Plenty Rd & Kingsbury Dr, Bundoora, VIC 3083 Australia

**Keywords:** Perinatal depression, Perinatal anxiety, Perinatal mental illness, Telephone helpline, Volunteer, Peer support, Telephone counselling

## Abstract

**Background:**

Anxiety and depression are common in the perinatal period. Telephone interventions, including telephone peer support and counselling, have been developed to support those experiencing perinatal mental illness. PANDA Post and Antenatal Depression Association provides support to women and men experiencing perinatal mental illness via the Australian National Perinatal Depression Helpline, encompassing both volunteer peer support and professional counselling. This study aimed to explore the experiences of callers to the Helpline.

**Methods:**

A cross-sectional survey design was used. All new callers from 1^st^ May to 30^th^ September 2013 were invited to participate. The survey, adapted from a previous survey of PANDA callers, included 23 questions using Likert-type scales, demographic and open-ended questions. Thematic network analysis was undertaken for responses to open-ended questions.

**Results:**

124 responses were received (124/405; 30 % response). The majority of callers had called the Helpline regarding themselves (90 %), with over one third (33 %) of all callers seeking crisis support and help. Ninety-nine per cent of respondents ‘agreed’ or ‘strongly agreed’ that staff and/or volunteers understood their concerns, and 97 % ‘agreed’ or ‘strongly agreed’ that overall PANDA had helped them. Callers described the PANDA service as uniquely tailored to the perinatal period, providing accessible, non-judgemental understanding and support, with a global theme from open-ended comments describing PANDA as ‘a safe space to be heard and receive support without judgement’. Recommendations for service changes included increased hours of availability.

**Conclusions:**

Callers reported positive experiences of accessing support from the PANDA National Perinatal Depression Helpline. The Helpline was described as an accessible and acceptable telephone support for individuals experiencing perinatal mental illness. Recommendations for changes to the service included an increase in hours of operation to enable greater responsiveness at times of need, reduced waiting times, and access to continuity with the same volunteer and/or telephone counsellor. The findings of the study will be useful in informing future service provision, review, and implementation.

## Background

Perinatal mental illness encompasses mental health disorders occurring during pregnancy and the first year after birth, including depression, anxiety disorders and postpartum psychosis [1]. In Australia perinatal mental illness is a leading cause of indirect maternal death [2].

Although the prevalence of perinatal mental illness varies in different studies [1], estimates indicate around 18 % of women will experience depression during their pregnancy [3], and between 13 and 19 % in the first year after birth [3–8]. Approximately 10 % of men may also experience depression during their partner’s pregnancy and/or in the first year after birth [9]. Perinatal anxiety is less commonly researched than depression, but is estimated to affect between 10 to 16 % of women and 4 to 10 % of men in the postpartum period [10].

A wide range of pharmacological, psychosocial, psychological, and complementary and alternative therapies have been used to treat perinatal mental illness [1], including interpersonal psychotherapy, cognitive-behavioural therapy [11], antidepressant medication [12], psychosocial interventions [13], and exercise such as yoga [14]. However, for services supporting those experiencing perinatal mental illness to be accessible and acceptable, understanding is required of both the help-seeking behaviours for, and the facilitators and barriers to, support and treatment. A qualitative systematic review including 40 studies, some of which included women from diverse cultural backgrounds, explored the help-seeking barriers and facilitators for women with postnatal depression. The study identified that many women wanted ‘to be given permission to talk in-depth about their feelings… [and have] recognition that there was a problem and reassurance that other mothers experience similar feelings and that they would get better’ [15] (p.327). Barriers to women seeking help included inconvenience of attending appointments, insufficient time, and a lack of awareness of postpartum depression [15]. A study exploring help-seeking for anxiety and depression after childbirth conducted in Australia found that women experiencing anxiety were less likely to seek help from a health professional compared to women experiencing depression or both anxiety and depression [16]. Women who did not seek help most commonly cited being able to deal with the problem themselves; being too busy or having not yet ‘got around’ to seeking help; not having anyone they were comfortable to talk to; or feeling embarrassed as reasons they did not access help from a health professional [16] (p.81).

## Telephone peer support and counselling

A number of telephone-based interventions have been used in maternity care [17], including volunteer peer support telephone hotlines for breastfeeding and perinatal mental health support [18]. Peer support within the health care context has been defined as ‘the provision of emotional, appraisal, and informational assistance by a created social network member who possesses experiential knowledge of a specific behaviour or stressor and similar characteristics as the target population, to address a health-related issue of a potentially or actually stressed focal person’ [19] (p.329).

Telephone-based health support has been identified as private and flexible [20], with the potential to overcome barriers including access to transport, fear of stigmatisation [20, 21], and geographical isolation [17, 22]. Telephone-based health care also appears to be acceptable, with a systematic review of telemedicine compared to face-to-face care finding that telemedicine was well accepted by recipients [23]. Dennis and Kingston however note that telephone support can be impacted by difficulties including language barriers, and that ‘support is less likely when recipients are required to initiate telephone contact compared with provider initiated services’ [20] (p. 301). A 2008 systematic review of telephone support for women during pregnancy and the early postpartum period noted that although the number of telephone interventions in pregnancy and postpartum had increased significantly, there has been limited research evaluating these interventions, and few of the studies conducted included a maternal evaluation of the intervention [20].

A small number of studies have explored the effectiveness of telephone support, including telephone peer support, to prevent or treat maternal postnatal depression and anxiety [24–26]. A multi-site randomised controlled trial (RCT) [24] explored the effect of proactive individualised telephone peer support on the prevention of postnatal depression among women identified as being at high risk of developing postnatal depression. Fourteen per cent of women in the intervention group had an Edinburgh Postnatal Depression Score >12 at 12 weeks postpartum, a score indicating probable major depression [27], compared with 25 % of women in the control group. Maternal satisfaction with the intervention was high, with over 80 % of women satisfied with their experience and stating that they would recommend this type of support to a friend [28].

A 2014 qualitative systematic review explored women’s experiences of peer support for perinatal mental illness [29]. Five relevant studies were identified, with peer support received in the form of a peer support group in all studies. The authors identified four themes from the meta-ethnography: isolation: the role of peer support; seeking validation through peer support; the importance of social norms of motherhood, and finding affirmation/a way forward; and the impact of peer support. Women in the studies identified feeling isolated, with some highlighting that their feelings of isolation were further heightened when other mothers did not share their experiences of perinatal mental illness. The authors found that women had a need to discuss their thoughts and feelings with someone as a way of reducing their distress. Many women were seeking validation, and the authors note that when women ‘encountered others who validated their feelings and their parenting experiences, life became less difficult’ [29] (p.495).

## PANDA Post and Antenatal Depression Association

PANDA Post and Antenatal Depression Association began in the 1980s as a peer support organisation in Victoria, Australia [30]. PANDA provides support to the Australian community in multiple ways, including online fact sheets, two websites, and a National Perinatal Depression Helpline, hereafter referred to as the Helpline, which was launched in July 2010. The helpline is available to callers Monday to Friday from 10am to 5pm, with more than ten thousand calls made to and from the Helpline each year. The majority of callers to the Helpline are women [31]. Callers hear of the PANDA Helpline through different pathways, including midwives and other maternity care providers, general practitioners, maternal and child health nurses, the PANDA website, and word of mouth from friends and family.

Volunteers who provide peer support to callers and counsellors who provide professional counselling services staff the Helpline. PANDA recruits and trains volunteer peer support workers for the Helpline and also for home visiting and community education activities. Volunteers are recruited through word of mouth, the PANDA website and online volunteer opportunity websites. Volunteers have experienced, or have supported someone who has experienced perinatal mental illness. Some volunteers join PANDA as they have a professional interest in supporting individuals experiencing perinatal mental illness. Individuals interested in volunteering with PANDA apply and attend an information session to better understand what the role would entail. Prospective volunteers also undergo an individual interview, police and referee checks. Successful telephone support volunteer applicants then undertake a two stage training process; stage one involves 24 h of group education delivered over an eight week period, and stage two includes observing experienced volunteers and/or counsellors on the Helpline until the individual volunteer displays readiness to conduct their first phone call. The education provided to volunteers focuses on perinatal mental health, loss and grief, transition to parenthood, family of origin, attachment theory, the Helpline systems and processes, counselling skills, risk assessment, values and self-care. All volunteers on the Helpline are required to complete a two day Applied Suicide Intervention Skills training (ASIST). A volunteer coordinator is present to support volunteers on the Helpline at all times. Professionally trained counsellors are also employed by the organisation to provide telephone counselling as part of the Helpline services. At the time of the evaluation there were a total of 40 telephone support volunteers and 12 telephone counsellors working on the Helpline.

**Fig. 1:**
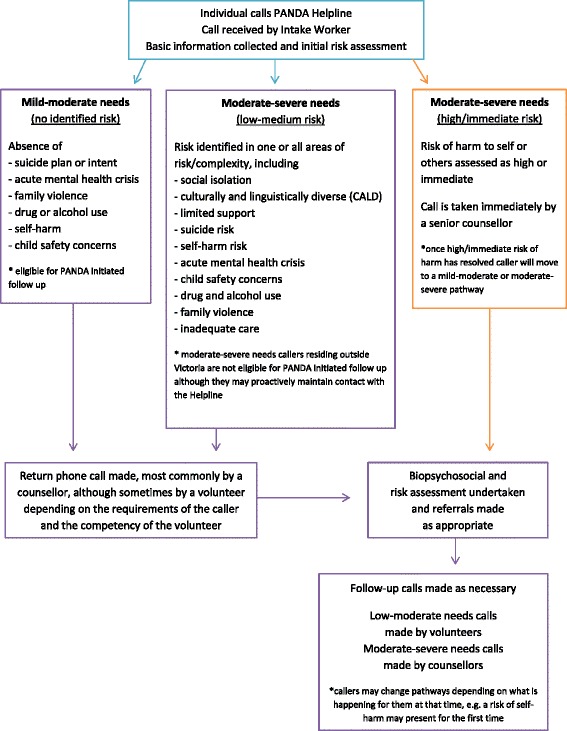
Risk assessment and pathways

Incoming calls to the Helpline are answered by the Intake Worker who will record some initial information and conduct an initial risk assessment (Fig. 1). Unless there are immediate concerns regarding the caller’s safety they will receive a phone call back from a PANDA volunteer or telephone counsellor, depending on the caller’s needs, to enable a thorough initial call to be undertaken. This call back is usually undertaken the same day the individual makes contact with the Helpline. It is common at the end of this first call for the PANDA volunteer or counsellor to seek permission for PANDA to initiate follow-up support calls to ensure callers are accessing supports in their local community.

Due to different funding structures callers assessed as ‘moderate to severe’ needs residing outside the state of Victoria are not eligible to receive PANDA initiated follow-up calls once they are considered to be ‘linked’ with a local service provider, such as a GP, however callers can contact the Helpline if they require support.

In 2013 an independent evaluation of the PANDA Helpline was undertaken. The evaluation included four components: a description of the PANDA caller profile, demand and referral pathways; an exploration of the views and experiences of callers to the PANDA Helpline; telephone interviews with callers assessed as moderate-severe needs; and key informant consultations with PANDA staff, volunteers, and key stakeholders. The component of the evaluation that explored the views and experiences of callers to the Helpline is the focus of this paper.

## Methods

### Aims

This study used a cross-sectional survey design to explore callers’ views and experiences of their contact with PANDA, including interactions with staff and/or volunteers, their reason(s) for calling the service, overall experiences of care, and their views of how the service was organised.

Specifically, the study aimed to explore:

- callers’ experiences of communication with PANDA, including why they called the Helpline, and what they felt they had gained from their contact with the service;
- how, if at all, the Helpline differed from other support services callers had accessed; and
- if there were things that could be improved within the PANDA service.

All new callers^1^ to PANDA, from 1^st^ May to 30^th^ September 2013 were invited to participate in the survey four to eight weeks after their initial contact. Callers’ contact details, such as an email or postal address, were obtained by PANDA staff and/or volunteers during their first call as part of ‘usual care’ on the Helpline. Callers were contacted four to eight weeks after the initial contact as it was anticipated that most would no longer be in contact with PANDA and that the timeframe was recent enough for accurate recall. Sample size calculations were not performed as the survey was part of a larger service evaluation and was designed and timed to maximise the number of possible participants while also meeting the time constraints of the overall evaluation.

### Survey instrument

The survey was adapted from a previous survey of PANDA callers conducted as part of a Master’s study in 2012 [32]. The adapted survey was piloted with experienced maternity clinicians and perinatal researchers who reviewed the survey for content, flow, face and content validity. The survey consisted of 23 questions, and included a range of closed and open-ended questions, as well as a number of statements with Likert-type scale response options, i.e., ‘strongly agree’, ‘agree’, ‘neither’, ‘disagree’ or ‘strongly disagree’. Demographic questions were also included. The questions explored how callers had heard of PANDA, number of contacts, overall experiences with the Helpline, call content, views regarding organisational features, referrals to other support services, and suggestions for changes to the Helpline.

### Data collection

Surveys were sent to all new callers within the study period by email if PANDA had email details or by hard copy with a reply paid envelope if only a postal address was available. The surveys were sent by PANDA and received by the research team; therefore, the research team had no knowledge of the caller’s identity, and the PANDA staff had no access to callers’ responses. A detailed letter was sent with the invitation explaining the purpose of the study. The letter explained that all responses would remain anonymous, that they would be sent directly to the research team, and a decision not to participate or any responses provided would not impact on any current or future contact with the Helpline. Callers were sent two reminders using the same method (either email or postal) that they had initially been contacted with, one two weeks after the initial contact and the second two weeks after the first reminder. As all responses were anonymous it was not possible to send reminders to only those who had not completed the survey, and therefore a note was included thanking those who had already responded for their time. Return of the survey was taken as consent to participate.

### Analysis

All data were collected and managed within the secure web-based application Research Electronic Data Capture (REDCap) [33], with hard-copy surveys entered directly into the application by one of the research team. Data were entered at the time of the evaluation by a member of the research team, and checked by LB. No errors were identified. Data cleaning included range and logic checks. Data analysis using descriptive statistics was undertaken within STATA 11 [34], and results mostly presented as numbers and per centages. Some survey questions were not asked if a respondent indicated that they had contacted the Helpline for someone other than themselves, such as a partner or family member. As a result some data presented are for respondents who called for themselves only; this is made explicit within tables as ‘called regarding self’ or ‘called regarding partner/other’. Open-ended questions were asked of all survey respondents and analysis has included all responses.

Responses to open-ended questions were analysed thematically [35], with both LB and HMcL undertaking qualitative data analysis. The thematic analysis has been presented as a thematic network, a ‘web-like’ illustration which aims to present the key themes within the text and the relationships between them [36] (p.386). The network has three levels of themes: basic themes made up of lowest-order premises; groups of basic themes summarising more abstract principles, known as organising themes; and global themes which encompass the principal metaphors within the text [36].

Some respondents did not answer every question within the survey, and so for some questions the denominator changes. This has been made explicit within tables with each question presented with its own denominator.

Ethics approval was obtained from the La Trobe University Ethics Committee, application number FHEC11/057.

## Results

**Fig. 2:**
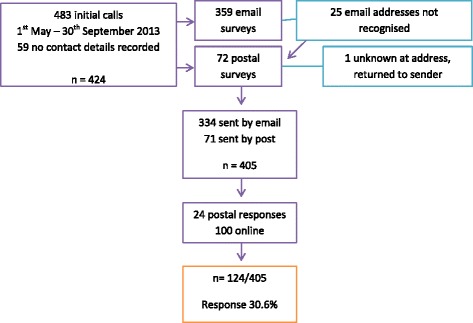
Participant flow chart

Figure 2 describes the process of distributing the survey to callers and the responses received. A total of 483 new calls were made to the Helpline over the five-month recruitment period, but 59 callers did not provide any contact details. In total 359 emails and 72 postal surveys (n = 431) were sent. Of the original 359 sent by email, 25 of the email addresses were incorrect. Seven of these had postal addresses, so this method was used in these instances. One postal survey was ‘returned to sender’ with an incorrect address. Thus, there was potential for 405 surveys to be completed and of these 124 responses were received, 24 hard copy and 100 via the online survey; a response of 30 %.

### Characteristics of respondents

Of the 124 responses received, 112 (90 %) had called PANDA regarding themselves, and six regarding their partner, three for their sister and one for their daughter.

Respondents who called regarding themselves were mostly female (97 %), married (78 %) or living with a partner (17 %), born in Australia (75 %), living in the state of Victoria (50 %), spoke English as a first language (87 %), and had completed a university degree or higher (60 %) (Table 1).

**Table 1:** Characteristics of participants (calling for self or calling for partner)

	Called regarding self		Called regarding partner^b^	Total	
	n	%	n	n	%
Sex	(n = 99)		(n = 5)	(n = 104)	
ᅟFemale	96	97.0	3	99	95.2
ᅟMale	3	3.0	2	5	4.8
Caller age in years	(n = 98)		(n = 5)	(n = 103)	
ᅟ<25	5	5.1	0	5	4.9
ᅟ25-30	21	21.4	2	23	22.3
ᅟ30-40	69	70.4	3	72	69.9
ᅟ>40	3	3.1	0	3	2.9
Marital status	(n = 99)		(n = 5)	(n = 104)	
ᅟMarried	78	78.8	3	81	77.9
ᅟLiving with a partner	17	17.2	2	19	18.3
ᅟSeparated or divorced	2	2.0	0	2	2.0
ᅟHave a partner but do not live together	1	1.0	0	1	1.0
ᅟSingle	1	1.0	0	1	1.0
ᅟOther	0	0.0	0	0	0.0
Number of children	(n = 98)		(n = 5)	(n = 103)	
ᅟNone	9	9.2	0	9	8.7
ᅟOne child	48	49.0	4	52	50.5
ᅟTwo children	37	37.7	1	38	36.9
ᅟThree children	4	4.1	0	4	3.9
Age of most recent child^a^	(n = 93)		(n = 5)	(n = 98)	
ᅟ≤12 weeks	10	10.7	0	10	10.2
ᅟ13-26 weeks	29	31.2	3	32	32.6
ᅟ27-51 weeks	33	35.5	2	35	35.7
ᅟ≥52 weeks	21	22.6	0	21	21.4
Pregnant at time of survey	(n = 99)				
	16	16.2			
Education	(n = 99)		(n = 5)	(n = 104)	
ᅟCompleted a degree or higher	60	60.6	3	63	60.6
ᅟCompleted a certificate/diploma/ apprenticeship	31	31.3	1	32	30.8
ᅟCompleted secondary school Year 12	5	5.0	1	6	5.8
ᅟHave not completed secondary school	3	3.0	0	3	2.9
Total before tax household income/week (AUD)	(n = 98)		(n = 5)	(n = 103)	
ᅟLess than $650	6	6.1	0	6	5.8
ᅟ$650 - $999	13	13.3	0	13	12.6
ᅟ$1000 - $1399	17	17.3	1	18	17.5
ᅟ$1400 - 1999	27	27.5	2	29	28.1
ᅟ> $2000	35	35.7	2	37	35.9
Pension the main family income	(n = 98)				
	4	4.0			
State of residence	(n = 99)		(n = 5)	(n = 104)	
ᅟVictoria	50	50.5	4	54	51.9
ᅟNew South Wales	18	18.2	1	19	18.3
ᅟQueensland	17	17.2	0	17	16.3
ᅟSouth Australia	9	9.1	0	9	8.6
ᅟWestern Australia	4	4.0	0	4	3.8
ᅟAustralian Capital Territory	1	1.0	0	1	1.0
ᅟTasmania	0	0.0	0	0	0
ᅟNorthern Territory	0	0.0	0	0	0
Aboriginal or Torres Strait Islander origin	(n = 98)		(n = 5)	(n = 103)	
ᅟAboriginal	1	1.0	0	1	1.0
ᅟTorres Strait Islander	0	0.0	0	0	0.0
Country of Birth	(n = 98)		(n = 5)	(n = 103)	
ᅟAustralia	74	75.5	4	78	75.7
Years since settlement in Australia (non-Australian born)	(n = 23)		(n = 1)	(n = 24)	
ᅟ< 5 years	5	21.7	0	5	20.8
First language	(n = 96)		(n = 5)	(n = 101)	
ᅟEnglish	84	87.5	5	89	88.1

^a^Only those who reported having a child ^b^per centage not supplied due to small numbers

Respondents who called for someone other than themselves were mostly female (77 %), married or living with a partner (100 %), born in Australia (77 %), and spoke English as a first language (100 %).

### Contacting PANDA

Table 2 details the reasons respondents had called PANDA; callers were most commonly seeking support (76 %), information about postnatal depression and anxiety (44 %), and reassurance (41 %). Of those who had called PANDA for themselves, 33 % were seeking crisis support. Eighty-six (76 %) callers reported that PANDA suggested they contact other support services, and of those 74 (88 %) reported that they were able to access these services.

**Table 2:** Reasons respondents contacted PANDA and feelings before making first call

	Called regarding self		Called regarding other^b^
Reasons for contact	(n = 112)	%^a^	(n = 10)
ᅟSupport	86	76.8	7
ᅟInformation about postnatal depression and anxiety	50	44.6	5
ᅟReassurance	46	41.1	5
ᅟCrisis support/help	37	33.0	2
ᅟReferral to other services	17	15.2	3
ᅟInformation about antenatal depression and anxiety	16	14.3	2
Feelings before making first call to PANDA	(n = 112)	%^a^	
ᅟDesperate	72	64.3	
ᅟNervous	52	46.4	
ᅟUnsure of how PANDA could help	51	45.5	
ᅟScared	42	37.5	
ᅟHopeful	20	17.8	
ᅟOptimistic	5	4.5	
ᅟRelieved	3	2.7	
Other (e.g., anxious, worried)	10	8.9	

^a^Respondents could make more than one selection ^b^per centage not supplied due to small numbers

Callers who had accessed PANDA for themselves were asked how they felt before picking up the phone and making their first call. Respondents most commonly reported feeling desperate (64 %), nervous (46 %), unsure of how PANDA could help (45 %), and scared (37 %) (Table 2).

Respondents were asked to estimate how many calls they had made to PANDA, and also how many calls PANDA had made to them (or how many messages PANDA had left) (Table 3). Half of the respondents reported making one call to the Helpline (50 %), and most (89 %) received PANDA initiated follow-up, in the form of phone calls or messages. Of those who did receive calls from PANDA, more than a third (34 %) received five or more phone calls.

**Table 3:** Frequency of contact with PANDA (Called regarding self and other)

	n	%
Calls made to PANDA (n = 122)		
ᅟ1	61	50.0
ᅟ2	29	23.8
ᅟ3-4	20	16.4
ᅟ≥5	11	9.0
Did PANDA make calls or leave messages to you (n = 123)		
ᅟYes	110	89.4
Number of calls made by PANDA (n = 109)		
ᅟ≤2	43	39.4
ᅟ3-4	28	25.7
ᅟ≥5	38	34.9
Number of messages left by PANDA (n = 88)		
ᅟ≤2	59	67.0
ᅟ3-4	20	22.7
ᅟ≥5	9	10.2

### Call content

Respondents were given a list of topics to respond to regarding what was discussed or provided to them during their call/s with PANDA (Table 4). All callers responded that PANDA staff and/or volunteers had asked them how they were feeling and listened to their story. All except one respondent reported being provided with information they could trust (99 %) and being asked about their relationships (99 %). The majority of callers (97 %) felt that they gained hope and reassurance, and were helped to feel better about being a parent (97 %). Callers also responded that they were supported to help identify what they were doing well and to develop realistic views of what is possible (88 %); to understand the impact of early childhood or young adult life experiences on parenting (65 %); and that they were provided with referrals to other services (83 %). Safety was an important feature of most conversations, with most identifying that they were asked by PANDA staff and/or volunteers if they felt safe (94 %) and if their baby/children were safe (93 %), and most reported that staff and/or volunteers had acted to keep them or their baby/children safe (81 %).

**Table 4:** Call content (Called regarding self)

The PANDA staff/volunteers:	n^a^	%
Asked me how I was feeling (n = 105)	105	100.0
Listened to my story (n = 105)	105	100.0
Asked me about my relationship (n = 103)	102	99.0
Provided me with information I could trust (n = 104)	103	99.0
Reassured me and gave me hope (n = 106)	103	97.2
Helped me to feel better about being a parent (n = 99)	96	97.0
Followed up on things from previous calls (n = 89)	86	96.6
Made sure I was seeing my health care providers (n = 103)	99	96.1
Asked me if I was feeling safe (n = 94)	89	94.7
Asked me if my baby/children were safe (n = 89)	83	93.3
Discussed strategies to manage my distress (n = 102)	95	93.1
Encouraged me to talk to my partner/family about experiences (n = 95)	85	89.5
Provided me with referrals to other services (n = 92)	77	83.7
Acted to keep me or my baby/children safe (n = 53)	43	81.1
Encouraged me to spend time connecting with my baby (n = 79)	58	73.4
Sent me an information pack (n = 99)	72	72.7
Encouraged me to get an accurate diagnosis (n = 83)	60	72.3
Asked me about my experience of the birth (n = 85)	60	70.6
Supported people who were helping me (n = 60)	25	41.7
Offered to talk to my health care providers (n = 80)	23	28.7
Helped me to identify what I am doing well and develop realistic views of what is possible (n = 98)	87	88.8
Assisted me to understand the impact of my life as a child and young adult on my experience of becoming a parent (n = 87)	57	65.5

^a^These questions could be answered as ‘yes’ or ‘no’. The n provided above is the number of respondents who answered the question, and the n and % in the columns indicates the number and per centage who answered ‘yes’

### Views and experiences of callers to PANDA

Callers were asked a set of questions regarding their views and experiences of their contact with PANDA. Ninety-nine per cent of respondents ‘agreed’ or ‘strongly agreed’ (hereafter referred to as agreed) that PANDA staff and volunteers understood their concerns, and 94 % agreed that they felt emotionally better after speaking with someone from PANDA (Table 5). Over 99 % of respondents agreed that PANDA provided non-judgemental support, and a total of 97 % agreed that overall PANDA had helped them.

**Table 5:** Views and experiences of callers to PANDA (all participants)

	Strongly Disagree		Disagree		Neither		Agree		Strongly Agree	
	n	%	n	%	n	%	n	%	n	%
The PANDA staff/volunteers were relaxed and unhurried on the phone (n = 118)	0	0.0	1	0.8	0	0.0	19	16.1	98	83.0
The PANDA staff/volunteers provided non-judgemental support (n = 117)	0	0.0	0	0.0	1	0.8	21	17.9	95	81.2
Overall, PANDA helped me (n = 116)	0	0.0	1	0.9	2	1.7	19	16.4	94	81.0
I always felt my worries, anxieties or concerns were taken seriously by the PANDA staff/volunteers (n = 117)	1	0.8	0	0.0	2	1.7	21	17.9	93	79.5
The PANDA staff/volunteers understood my concerns (n = 118)	0	0.0	0	0.0	1	0.8	24	20.3	93	78.8
I was happy with the emotional support I received from PANDA staff/volunteers (n = 117)	0	0.0	0	0.0	4	3.4	27	23.1	86	73.5
The PANDA staff/volunteers were encouraging and reassuring (n = 118)	0	0.0	1	0.8	1	0.8	30	25.4	86	72.9
Emotionally, I felt much better after speaking with someone from PANDA (n = 118)	0	0.0	2	1.7	4	3.4	36	30.5	76	64.4
The PANDA staff/volunteers helped me to understand antenatal and/or postnatal depression and anxiety (n = 118)	0	0.0	4	3.4	16	13.5	39	33.0	59	50.0
During the phone conversations with PANDA, I was always asked whether I had any questions (n = 118)	1	0.8	1	0.8	16	13.5	53	44.9	47	39.8
I was NOT happy with the information given to me by the PANDA staff/volunteers (n = 118)	91	77.1	20	16.9	6	5.1	0	0.0	1	0.8
Having access to PANDA during the evening/night and at weekends is important (n = 117)	0	0.0	0	0.0	4	3.4	40	34.2	73	62.4
It is important to be able to speak to the same person during every phone call with PANDA (n = 118)	3	2.5	24	20.3	27	22.9	42	35.6	22	18.6
I was comfortable leaving my contact details with PANDA so that they could call me back (n = 117)	0	0.0	2	1.7	3	2.6	38	32.5	74	63.2
It is important that the person I talk to at PANDA has personally experienced antenatal and/or postnatal depression or anxiety (n = 118)	9	7.6	37	31.3	38	32.2	26	22.0	8	6.8
It took a long time for a counsellor to return my call (n = 117)	48	41.0	45	38.5	9	7.7	12	10.2	3	2.6
I would have liked the option to have someone from PANDA visit me at home AS WELL AS receiving support by telephone (n = 117)	6	5.1	19	16.2	27	23.1	42	35.9	23	19.6

Callers were also asked a series of questions regarding what features they considered important for PANDA to offer as an organisation (Table 5). Ninety-six per cent of callers agreed that having access to PANDA during the evening/night and at weekends was important. When asked whether it was important that the person they spoke to at PANDA had personal experience of antenatal and/or postnatal depression or anxiety, this was one of the few areas of mixed response, with 28 % agreeing or strongly agreeing it was important, 38 % disagreeing or strongly disagreeing, and the remaining 32 % neither agreeing nor disagreeing. Responses regarding the option of having someone from PANDA visit them at home as well as receiving telephone support also received mixed response, with 19 % of callers strongly agreeing that they would like this option, 35 % agreeing, 23 % neither agreeing nor disagreeing, and 21 % disagreeing or strongly disagreeing with the statement. Eighteen per cent strongly agreed that it was important to be able to speak to the same person during every phone call, 35 % agreed, 22 % neither agreed nor disagreed, and 22 % disagreed or strongly disagreed that this was important to them.

### Exploring what callers had gained from their contact with PANDA

**Fig. 3:**
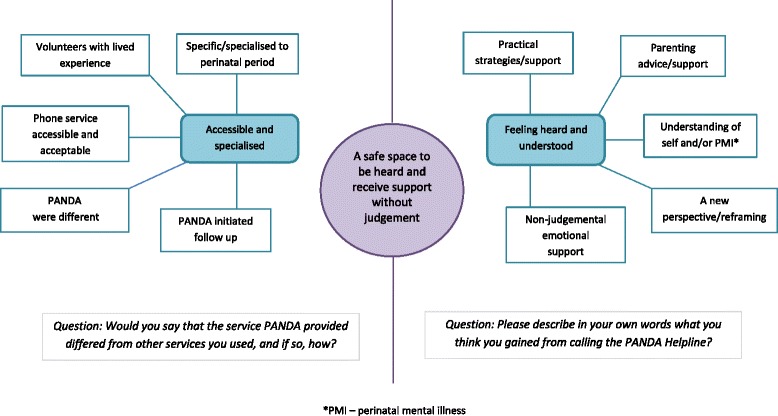
Thematic network for analysis of two open-ended questions

Thematic analysis [35] was undertaken with responses to two open-ended questions: *‘Would you say that the service PANDA provided differed from other services you used, and if so, how?’* (83 responses received) and *‘Please describe in your own words what you think you gained from calling the PANDA Helpline?’* (98 responses received). Analyses have been presented as a thematic network [36] incorporating 11 basic themes, two organising themes and one global theme (Fig. 3).

### Thematic network

During early data analysis it became clear that there were commonalities between answers to both questions, which led to the decision to present the analyses as a thematic network; recognising the overall picture the two analyses were able to create whilst keeping the two sides of the network, each originating from responses to different questions separate. The global theme **a safe space to be heard and receive support without judgement** has been used to describe the thematic network. Callers’ responses describe the PANDA service as unique, accessible and acceptable. Respondents felt that PANDA created a safe space where they could discuss private and complex emotions without fear of judgement. Callers felt that they had been properly heard and understood by the staff and/or volunteers, that their experiences were validated, and that they had received both emotional and practical support to begin to address their individual situation.

*Question 1: ‘Would you say that the service PANDA provided differed from other services you used, and if so, how?’*

The organising theme **‘accessible and specialised’** was developed from five basic themes incorporating callers’ descriptions of PANDA’s service and how, if at all, it differed from other services they had used: specific/specialised to the perinatal period; volunteers with lived experience; phone service accessible and acceptable; PANDA was different; PANDA initiated follow up.

Callers described PANDA as a service **specific/specialised to the perinatal period**, with a unique, high level of knowledge regarding both maternal and paternal mental health at this time:

*‘The fact that PANDA speciali[s]es in post natal/pre natal conditions seems to have made all the difference.’* (Participant 96 - 34 year old woman, called regarding herself)

The benefit of being able to speak to someone who has a **lived experience of perinatal mental illness** was highlighted, with callers describing this as contributing to them feeling heard and understood:

*‘It was fantastic to talk to someone to who had been through PND. It makes a difference to know they understand how you are feeling.’* (Participant 52 - 35 year old woman, called regarding herself)

*‘Having volunteers that have experience themselves.’* (Participant 34 – 27 year old woman, called regarding herself)

Callers emphasised the increased **accessibility and acceptability of the telephone service**, describing it as easy, relaxed and not rushed, anonymous, and available and responsive at the time of need, as it was not appointment based:

*‘PANDA was at the end of the phone line when I needed them - and I could call them crying from the privacy of my home, in my daggy clothes (therefore different to having to make an appointment sometime in the future with a counsellor, or go out in public to see my GP).’* (Participant 54 – 37 year old woman, called regarding herself)

*‘I felt ashamed so non face to face contact was good for me in the beginning…’* (Participant 11 – 30 year old woman, called regarding self)

Callers made some explicit comments regarding how PANDA **was** different to other specific services, describing less positive experiences with these services:

*‘Other general services I have called have belittled the problems and feelings associated with parenting and made the call as short as possible to get rid of me, telling me to go to my GP.’ (*Participant 22 – 37 year old woman, called regarding herself)

*‘I have been to the doctor, seen a Councillor [sic], rang [another telephone service] numerous times and it wasn't until I spoke to PANDA that I felt like I was getting anywhere.’* (Participant 96 – 34 year old woman, called regarding herself)

Callers highlighted how important it was to them that **PANDA initiated follow up calls**, and that the responsibility was not always on them to initiate contact and ask for help and support:

*‘Telephone counselling was so useful as I was****completely****bedridden. I felt so ashamed of being sick/depressed so that****they****reached out to me with follow up phone calls was helpful’* (woman’s own emphasis - Participant 129 – 34 year old woman, called regarding self)

*‘The offer of regular follow-up is vital as depressed people, myself included, often self-isolate.’* (Participant 88 – 37 year old woman, called regarding herself)

*Question 2: ‘Please describe in your own words what you think you gained from calling the PANDA Helpline?’*

Callers’ descriptions of what they had gained from calling the Helpline led to the second organising theme **‘feeling heard and understood’**, which includes five basic themes: a new perspective/reframing; understanding of self and/or perinatal mental illness; practical strategies/support; parenting advice/support; and non-judgemental emotional support.

Respondents discussed the ways that their contact with PANDA had helped them to gain **new perspectives and reframe** their understanding, including helping them to gain a new or more balanced view of their situation. Callers made specific reference to the way PANDA helped them to focus on what they had achieved, rather than focusing on what they felt they had not:

*‘… it was so great to hear someone praise me for what I had been able to achieve, rather than (as I had been doing) focusing on what I felt I wasn’t doing well.’* (Participant 16 – 33 year old woman, called regarding herself)

*‘Helped me gain perspective on what I was achieving rather than what I was expecting myself to achieve.’* (Participant 50 – 28 year old woman, called regarding herself)

*‘…reframed my negative thoughts, gave me hope.’* (Participant 73 – 32 year old woman, called regarding herself)

Callers also described the way PANDA helped them gain **a better understanding of self and/or perinatal mental illness,** be it their own experiences and situation or that of a partner/family member:

*‘Understanding of post natal depression and how to support my wife’* (Participant 104 – 30 year old man, called regarding his wife)

*‘Help with understanding my feelings & emotions. Understanding!’* (Participant 118 – 35 year old woman, called regarding herself)

Respondents described varied ways that PANDA provided **practical support and guidance** to assist callers on a day-to-day basis, including advice regarding how to manage situations of increased anxiety by using tools such as mindfulness techniques. PANDA also helped callers access local perinatal mental health resources and gave assistance regarding ongoing care planning and support, either for themselves or for the partner/family member they were calling regarding:

*‘Objective viewpoint, validation and practical advice on how to proceed with supporting my partner/encouraging him to seek help.’* (Participant 91 – 33 year old woman, called regarding her partner)

*‘Practical assistance in accessing an enhanced maternal and child health nurse who put in to place access to a mother baby unit which helped in my recovery.’* (Participant 87 – 32 year old woman, called regarding herself)

*‘Helped me with strategies to consider during moments of anxiety.’* (Participant 50 – 28 year old woman, called regarding herself)

Respondents describe PANDA as providing important **parenting advice and support,** with a unique understanding of the experience of becoming a parent and the ways this may impact emotional wellbeing. Callers felt reassured that they were a good parent, gained confidence in their ability to parent well, and accessed practical, day to day parenting advice and support:

*‘Helped me maintain parenting confidence and [recognise] my strengths and that I am doing a great job despite other [people’s] judgements* (Participant 75 – 26 year old woman, called regarding herself)

*‘Reassurance that I was on the right track, ideas about how to tackle parenting problems’* (Participant 38 – 39 year old woman, called regarding herself)

Callers described the positive impact of the **non-judgemental emotional support** they received from PANDA, including feeling calmed, supported, validated, understood and not judged. They were able to feel that they were not beyond help, that their issues were genuine and that other women had experienced the same thing:

*‘I was heard. I felt understood. I felt my experience was validated. That I wasn't alone that my experience wasn't odd or so unique that it couldn't be helped or that it was all my fault.* (Participant 15 – 35 year old woman, called regarding herself)

*‘Understanding, and a freedom to talk about feelings that are very personal without any judgement.’* (Participant 22 - 37 year old woman, called regarding herself)

### Suggestions for service change

Callers were asked in open-ended questions to outline any suggestions for improvements which could be made to the PANDA service (41 responses received), as well as whether there was anything they had hoped to gain from their contact with the service which they did not receive (20 responses received). Similarly to the closed-ended questions regarding what service features callers considered important, responses included increased hours of availability, access to a known volunteer and/or counsellor, reduced waiting times, possible access to face to face services and increased availability of PANDA initiated follow up.

Callers suggested increased hours of availability to include evening, overnight and weekend times, emphasising the barriers to accessing the service during operating times:

*‘More funding so you could provide 24/7 help to women and families dealing with this illness.’* (Participant 27 - 31 year old woman, called regarding herself)

*‘I couldn't even call after hours when the kids are in bed as the service is not running. I did not want to call when I was at work. I need to be 'together' and functioning at work … so I don't have a meltdown in my professional environment… There were only two little windows available and it would have been much better to have been able to speak to someone when I was available to ring.* (Participant 15 - 35 year old woman, called regarding herself)

Respondents suggested greater availability of PANDA staff in order to reduce waiting times:

*‘More staff, shorter waiting times…’* (Participant 25 - 33 year old woman, called regarding herself)

*‘They also need more people available to take calls when u (sic) need them, not when they are available to call you back, when the crisis has passed.’* (Participant 43 - 37 year old woman, called regarding herself)

Some callers would have liked to access continuity with the same volunteer and/or counsellor:

*‘The thought that the first counsellor you speak to is the same person who calls back/is always there is nice (but prob not possible!) only because that person earns your trust - you have to go through it all again with someone else.’* (Participant 107 - 38 year old woman, called regarding herself)

Callers suggested PANDA could develop face-to-face drop in spaces or home visiting services:

*‘It would be great to have a drop in centre for face to face counselling or the option of home visits.’* (Participant 35 - 32 year old woman, called regarding herself)

Some callers who were not eligible for PANDA initiated follow up calls highlighted increased availability of proactive follow up calls as important:

*‘I would have enjoyed having staff follow up with me, but I understood that that service was no longer being offered.’* (Participant 16 - 33 year old woman, called regarding herself)

## Discussion

Overall, callers reported very positive experiences of their contact with the PANDA National Perinatal Depression Helpline. The majority of callers reported feeling better emotionally after speaking with someone from PANDA. Respondents had initiated contact with PANDA for support, information, and reassurance, with over one third of callers seeking crisis help and support. The reasons callers had initiated contact with the Helpline align well with the focus of the organisation as a telephone Helpline offering information, support and referral services. The large difference between the number of respondents seeking information for postnatal depression and anxiety (44 %) compared to antenatal depression and anxiety (14 %) might in part be explained by the greater clinical and research focus on perinatal mental illness in the postpartum period compared with pregnancy [37], or PANDA’s origins as an organisation focusing on supporting women experiencing postnatal depression.

The majority of callers to the Helpline agreed that PANDA was able to provide them with non-judgemental support, information they could trust, and that overall their contact with the service helped them. This was also reflected strongly in the thematic network, with callers describing in depth the different ways PANDA was able to provide them with emotional, practical and parenting support that was accessible and acceptable, as well as what they perceived made the service unique to others they had accessed. The majority of callers reported being helped by PANDA to feel better about being a parent, as well as gaining reassurance and hope.

The high levels of satisfaction expressed by respondents has similarities with previous studies exploring women’s experiences of perinatal mental illness, including the emphasis women place on the importance of having others to discuss experiences with [29, 38], the importance of social connection [39], women’s preferences for ‘talking therapies’ [15], and women’s satisfaction with a telephone peer support intervention [28].

Although less than one third of callers considered it important that the person they talked to at PANDA had personally experienced antenatal and/or postnatal depression or anxiety, analysis of the open-ended responses suggested that for some callers speaking with someone with a lived experience of perinatal mental illness was something that made PANDA’s service different to other services callers had accessed, and something that respondents saw as facilitating them feeling understood and supported. The concept of peer support focuses on the peer having lived experience similar to those whom they are supporting [19], and this has been a central feature of peer support interventions to date [18, 24], however this was not seen as essential by all respondents in this study.

Callers’ recommendations for possible changes to how the PANDA service is organised emphasised the importance of PANDA being more available at times of need, including afternoon/evening and weekend availability. The majority of respondents agreed that they would have liked the option of having someone from PANDA visit them at home as well as receiving telephone support, and that it was important to them to be able to speak with the same staff member/volunteer on each phone call they made to the service.

### Strengths and limitations

Very few studies have explored the experiences of those accessing telephone supports for perinatal mental health support. The findings of this study expand our knowledge of this, and could be used to help design and review telephone support services.

The representativeness of the survey sample was assessed by comparing routinely collected demographic data on all new callers over the previous ten-month period (January-October 2013). Survey respondents were generally representative of all new callers to the Helpline January - October 2013; mostly female (91 %), married (78 %), between the ages of 30-40 (70 %), and from the state of Victoria (53 %) [31]. Due to the way perinatal statistics are reported in Australia, the only demographic characteristic that could be used to compare our sample directly with the whole population of birthing women in Australia is mean age with callers to PANDA older than the overall population (mean age 32.4 versus 30.1) [40]. Marital status data were only available for the state of Victoria. Women in our sample more likely to be married (78.1 % versus 69.1 %) than the overall childbearing population in Victoria in 2011 [41].

The callers who access the PANDA Helpline are mostly female, older, and generally have a high level of education and income. While this is not representative of the general birthing population within Australia, or of those who experience perinatal mental illness, it was the aim of this study to explore the experiences of those accessing the Helpline and our sample reflected these characteristics.

While there is a need for more research about men’s mental health in the perinatal period [42], this study could not contribute to what is known about fathers’ experiences of help-seeking for perinatal mental illness as so few men contacted PANDA in 2013, and only three responded to the survey. This might in part be explained by men being less likely to seek help for mental illness than women [43].

The response to the survey was similar to the survey of callers to the PANDA Helpline in 2012, which was 32 % [32]. However, this is somewhat lower than earlier surveys of new mothers in Victoria [44] and is reflective of a widespread reduction in responses in population-based studies noted in the literature [45]. The response from this population might be expected to be lower than in other studies considering those contacting PANDA are likely to be experiencing distress.

## Conclusion

Overall, respondents reported very positive experiences of accessing support from PANDA Post and Antenatal Depression Association National Perinatal Depression Helpline. Callers describe the PANDA service as unique, specifically tailored to provide support and care to individuals experiencing perinatal mental illness and their partners, friends and family. Respondents emphasised the accessibility, acceptability and value of the telephone service, and made recommendations for changes to the service including an increase in hours of operation to enable greater responsiveness at times of need, reduced waiting times, and access to continuity with the same volunteer and/or telephone counsellor.

The Helpline is an accessible and acceptable telephone support for individuals experiencing perinatal mental illness. The findings of the study will be useful in informing future service provision, review, and implementation.

## Abbreviations

PANDA: PANDA Post and Antenatal Depression Association
RCT: Randomised controlled trial

## Footnotes

1 Callers may have previously contacted PANDA but be considered ‘new’ if they are not currently being counselled or followed up by PANDA staff and/or volunteers at the time of their call. As a result some callers classified as ‘new’ may have previously accessed support through the Helpline but are now re-engaging with the service after a period of non-contact (i.e., having sought support in pregnancy and then re-engage with service after birth).
